# Predicting dental caries outcomes in young adults using machine learning approach

**DOI:** 10.1186/s12903-024-04294-7

**Published:** 2024-05-03

**Authors:** Chukwuebuka Ogwo, Grant Brown, John Warren, Daniel Caplan, Steven Levy

**Affiliations:** 1 Department of Oral Health Sciences, Temple University Maurice H Kornberg School of Dentistry, 3223 N Broad Street, L216, Philadelphia, PA 19131 US; 2 Department of Biostatistics, College of Public Health, The University of Iowa, Iowa City, IA 52242 US; 3 Department of Preventive and Community Dentistry, The University of Iowa College of Dentistry, 801 Newton Rd, Iowa City, IA 52242 US; 4 Department of Epidemiology, College of Public Health, The University of Iowa, Iowa City, IA 52242 US

**Keywords:** Machine learning, Dental, Caries, Longitudinal, Artificial intelliegence, Prediction

## Abstract

**Objectives:**

To predict the dental caries outcomes in young adults from a set of longitudinally-obtained predictor variables and identify the most important predictors using machine learning techniques.

**Methods:**

This study was conducted using the Iowa Fluoride Study dataset. The predictor variables - sex, mother’s education, family income, composite socio-economic status (SES), caries experience at ages 9, 13, and 17, and the cumulative estimates of risk and protective factors, including fluoride, dietary, and behavioral variables from ages 5–9, 9–13, 13–17, and 17–23 were used to predict the age 23 D_2+_MFS count. The following machine learning models (LASSO regression, generalized boosting machines (GBM), negative binomial (NegGLM), and extreme gradient boosting models (XGBOOST)) were compared under 5-fold cross validation with nested resampling techniques.

**Results:**

The prevalence of cavitated level caries experience at age 23 (mean D_2+_MFS count) was 4.75. The predictive analysis found LASSO to be the best performing model (compared to GBM, NegGLM, and XGBOOST), with a root mean square error (RMSE) of 0.70, and coefficient of determination (R^2^) of 0.44. After dichotomization of the predicted and observed values of the LASSO regression, the classification results showed accuracy, precision, recall, and ROC AUC of 83.7%, 85.9%, 93.1%, 68.2%, respectively. Previous caries experience at age 13 and age 17 and sugar-sweetened beverages intakes at age 13 and age 17 were found to be the four most important predictors of cavitated caries count at age 23.

**Conclusion:**

Our machine learning model showed high accuracy and precision in the prediction of caries in young adults from a longitudinally-obtained predictor variables. Our model could, in the future, after further development and validation with other diverse population data, be used by public health specialists and policy-makers as a screening tool to identify the risk of caries in young adults and apply more targeted interventions. However, data from a more diverse population are needed to improve the quality and generalizability of caries prediction.

**Supplementary Information:**

The online version contains supplementary material available at 10.1186/s12903-024-04294-7.

## Introduction

Dental caries is a chronic infectious disease that destroys tooth structure and has significant public health implications, including in young adults [1]. The etiology of dental caries is multifactorial, with the most central etiological factors being the cariogenic diet, the action of bacteria, susceptible tooth structure, and time [1].

Few studies have explored the prevalence of cavitated caries in young adults and associated etiological factors. Brown et al.’s study using two National Health and Nutrition Examination Surveys (NHANES I and NHANES III) data found the mean Decayed, Missing Filled, Surface (DMFS) score of 24.8 and 13.9 among participants aged 18 to 25 years in NHANES I and NHANES III, respectively [2]. Also, Ismail et al.‘s study using the 1982–1983 Hispanic Health and Nutrition Examination Survey (HHANES) found a total mean DMFT score of 6.0 among Mexican-Americans aged 18 to 24 [3]. Garcia-Cortes et al. [4]. saw a high caries prevalence of 86.3% (mean DMFT of 5.8) among aged 22 to 25 applicants to San Luis Potosi University, Mexico with females having significantly higher DMFT than males (4.3 ± 4.0 vs. 3.9 ± 3.8; *p* = 0.04). Drachev et al. [5]. found high caries prevalence (96.0%; mean DMFT of 7.6) among Russian students, with higher mean DMFT in high socioeconomic status (SES) students compared to low SES students. A cohort study of Swedish children following clinical and radiographic examinations (age 20 mean DFS = 5.8) showed that previous caries experience at a younger age (ages 3, 6, and 15) was associated with caries experience at age 20 (*p* < 0.05) [6]. Jamieson et al.‘s study on Australian Aboriginal young adults aged 16 to 20 found a mean DMFT of 4.8 and that sex and sweets intake were significantly associated with higher mean DMFT [7].

Given the multifactorial and complex etiology of caries, there is a need for studies that use robust predictive modeling techniques like machine learning (ML) to accurately identify the best predictors of caries from complex datasets. Supervised machine learning is a type of artificial intelligence used to predict the value of an outcome measure based on several input measures. An ideal ML model has a favorable bias-variance trade-off (i.e., no model underfitting or overfitting) [8]. It provides a robust approach for the identification and selection of the most important predictors, while avoiding convergence issues and some aspects of the curse of dimensionality (Hughes phenomenon) [9], common issues in traditional statistical modeling with a large number of variables.

There are substantial gaps in our understanding of the predictive effects of longitudinally-obtained dietary/behavioral and fluoride variables on caries outcomes, especially in young adulthood, which is one of the most active stages of life. Previous ML studies [10, 11] have focused on the prediction of caries outcomes in children and we found no studies on the prediction of dental caries in young adults with a very wide range of comprehensive and cumulative (childhood) exposure variables using a machine learning approach. The objectives of this study were: (1) to predict the dental caries outcome in young adults using machine learning techniques and (2) to identify the most important predictors of the dental caries outcome from a large set of sociodemographic, dietary, fluoride, and behavioral variables.

## Methods

This was a secondary analysis of data collected from ages 5 to 23 within the Iowa Fluoride Study (IFS), a prospective cohort study that completed data collection in February 2019. The recruitment of IFS participants was done in the post-partum wards of eight Iowa hospitals from March 1992 to February 1995 [12]. The participants had dental exams approximately every four years (except for ages 17 to 23, an interval of 6 years) and received oral health questionnaires every six months Approval for the IFS was obtained from the University of Iowa Institutional Review Board for all components and procedures before the initiation of the study and for each examination, with annual renewal, as well as review when any modifications were done [13] (Appendix II).

The IFS dental examinations were done by one of three trained and calibrated dentists using portable dental equipment and halogen headlights with ongoing inter-examiner reliability assessment [13]. After drying the teeth, a DenLite® mirror (Welsh-Allyn Medical Products, Inc., Skaneateles Falls, NY) was used to enhance lighting and provide transillumination. The examinations were based primarily on visualization only, without radiographs, however, gentle explorer probing was used to confirm scoring, when in doubt. They were performed either at the University of Iowa College of Dentistry (Iowa City, IA) or at remote locations (Waterloo, IA, and Des Moines, IA) for those who could not make it to Iowa City. Caries status of each surface was recorded as either sound (S), arrested (D_0_), non-cavitated (D_1_), or cavitated (D_2+_); those with restorations were recorded as filled (F); missing teeth due to caries were recorded as missing (M) surfaces; and dental sealants were recorded separately [13].

The inclusion criteria for these analyses were (1) completion of the dental exams at age 23 and (2) having sufficient cumulative exposure to trapezoidal AUC estimate data (see Appendix I) for at least 35 out of the 51 independent variables for the time periods from ages 5 to 9, 9 to 13, 13 to 17, and 17 to 23.

The primary outcome variable (age 23 cavitated caries (D_2+_MFS) count) was defined as the sum of decayed (D_2+_cavitated), missing (M), and filled (F) surfaces at age 23. A total of 51 independent variables were considered, including four sociodemographic variables and 47 other predictors (cumulative exposure) variables. The sociodemographic variables were sex, family income level, mother’s level of education, and composite SES, with the last three assessed with data from a questionnaire in 2007. The main predictor variables were the cumulative exposure AUC variables for the periods from ages 5 to 9, 9 to 13, 13 to 17, and 17 to 23. They were defined for daily brushing frequency category, daily fluoride intake from combined sources, concentration of fluoride from home water, and the beverage variables (daily sugar-free beverage intake (no sugar added), daily milk intake, daily 100% juice intake, daily sugar-sweetened beverages intake, frequency of sugar-free (water-based) beverages consumption, frequency of milk consumption, frequency of 100% juice consumption, and frequency of sugar-sweetened beverages consumption). Additional variables were dental caries experience at ages 9, 13, and 17. Details of the variable definitions are provided in Appendix II.

### Statistical analysis

#### Exploratory data analysis

Descriptive statistics were determined for the person-level age 23 D_2+_MFS count and all independent variables. Bivariate analyses were conducted to ascertain the relationships between the dependent variable and each of the 51 independent variables. Mann-Whitney U tests were used to explore the relationships between age 23 D_2+_MFS count and sex and brushing frequency category; Kruskal-Wallis tests were used to explore the relationships between age 23 D_2+_MFS count and family income, mother’s level of education, and composite SES. Spearman (Rho) correlation tests were conducted to assess the relationships between the age 23 D_2+_MFS count and each of the continuous independent variables (home fluoride concentration, total fluoride intake, and beverage variables). All statistical analyses were performed with R software version 4.1.2, with a two-tailed alpha level set at 0.05 for bivariate analyses.

### Multivariable predictive modeling

Multivariable predictive modeling was performed using four ML models - Least Absolute Shrinkage and Selection Operator (LASSO) regression [8], negative binomial regression, generalized boosting machines (GBM) [14, 15], and extreme gradient boosting (XGBOOST) [16] - using the MachineShop [17] package for R (see Appendix III for the description of LASSO, GBM and XGBOOST). These models were chosen because of their abilities to (1) perform well with high dimensional data, (2) perform variable selection, and (3) handle different data types and distributions with very few assumptions (Details in Appendix III).

#### Data preprocessing

Prior to fitting the ML models, the k-nearest neighbor (KNN) imputation technique was used to handle the remaining missing data for these participants [18]. Additional data preprocessing (scaling and normalization) of the data was performed using the recipes package in R [19].

#### Model fitting (training and testing)

The training and testing of all models were done using the nested resampling technique with 5-fold cross-validation, which consists of an inner resampling loop and an outer resampling loop for testing the model performance [20]. We chose the nested resampling technique due to its ability to use different portions of the data to iteratively perform training and testing, thereby obtaining an unbiased performance estimate. In the outer resampling loop, we had five training/test sets (each with an 80 to 20 ratio). On each of these outer training sets, we optimized the model by performing parameter tuning and feature selection on the inner resampling loop. The optimized models then were fitted on the outer training sets and their performances were evaluated on the outer test sets. This technique gives a more honest estimation of model performance, although it is computationally expensive [20]. These models then were optimized by tuning them using the TunedModel function in the MachineShop package and the tuning parameters were chosen using the cross-validation technique [17].

#### Model evaluation

Model performance was assessed using root mean square error (RMSE), mean absolute error (MAE), and the R-squared value (coefficient of determination). Lower RMSE and MAE values indicate better model performance, while a higher R-squared value indicates better model performance. The best-performing model was selected based on the RMSE and R^2^. However, MAE was defined to better understand the overall model performance. The metrics for model performance were obtained by averaging the scores obtained from nested resampling with 5-fold cross-validation.

For easier interpretability, the observed and predicted values from the selected best model were first discretized and then dichotomized into dental caries as Yes (if values were above zero, indicating cavitated caries) or No (if values were zero, indicating no caries present). The following metrics then were used to show the model performance: accuracy, receiver operating characteristics area under the curve (ROC AUC), positive predictive value (precision), and sensitivity (recall). Details of the codes are provided in Appendix X. This study was reported using both the STROBE (Appendix XI) and TRIPOD guidelines (Appendix XII).

## Results

There were 258 participants who fulfilled the inclusion criteria, with 41 participants (16%) having at least one imputed data point and 3,458 out of 18,126 data points (19%) imputed using the k-nearest neighbor technique. There was favorable tooth-level inter-examiner reliability, with kappa statistics of 0.73, 0.71, 0.77, and 0.82 at ages 9, 13, 17, and 23, respectively.

Table 1 summarizes the frequency distributions of the categorical predictor variables. 58% of participants were female, and 13% of the subjects’ family income levels were below $40,000, with 48% $80,000 and above. About 14%, 32%, and 54% of participants were in the lower, middle, and higher SES groups, respectively.

**Table 1:** Descriptive analyses of the categorical independent variables

	*N*	Categories	Frequency (%)
Family income*	250	Less than $40,000	38 (14.73)
		$40,000 - $59,999	43 (16.67)
		$60,000 - $79,999	47 (18.22)
		$80,000 or more	122 (47.29)
Mother’s education*	256	High school diploma or lower	25 (9.70)
		Some college	33 (12.79)
		2-year college degree	67 (25.97)
		4-year college degree	70 (27.13)
		Post-graduate or professional degree	61 (23.64)
Composite SES*	250	Lower	32 (12.40)
		Middle	78 (30.32)
		Higher	140 (54.26)
Sex	258	Female	150 (58.14)
		Male	108 (41.86)
Age 5 to 9 brushing frequency^$^	249	< 1.5 times per day	60 (24.106)
		>=1.5 times per day	189 (75.90)
Age 9 to 13 brushing frequency^$^	246	< 1.5 times per day	52 (21.14)
		>=1.5 times per day	194 (78.86)
Age 13 to 17 brushing frequency^$^	240	< 1.5 times per day	35 (14.58)
		>=1.5 times per day	205 (85.42)
Age 17 to 23 brushing frequency^$^	219	< 1.5 times per day	26 (11.87)
		>=1.5 times per day	193 (88.13)

Composite SES was defined based on the combination of two variables (mother’s educational level and family income (see Appendix II for details of the variable definition)

* Data were collected in 2007

^$^ Cumulative exposure variable based on trapezoidal AUC estimates and transformed into two categories

As shown in Table 2, the prevalence of cavitated caries at age 23 (D_2+_MFS_23_ count) was 69.1%, with a mean D_2+_MFS_23_ of 4.75 (SD = 4.32). The mean values for the cumulative exposure AUC predictor variables from ages 5 to 9, 9 to 13, 13 to 17, and 17 to 23 were: 0.71, 0.72, 0.85, and 1.04, respectively, for fluoride intake from combined sources (mg F/day); 1.67, 1.50, 1.56, and 1.11, respectively, for milk intake per day (cups/day); 0.61, 1.16, 1.42, and 1.76, respectively, for 100% juice intake (cups/day); and 0.61, 1.16, 1.42 and 1.76, respectively, for intake of sugar-sweetened beverages (cups/day). Also, mean caries (D_2+_MFS) experience at ages 9, 13, and 17 were 0.46, 1.15, and 2.94, respectively (See Appendix IV for more details about the descriptive statistics).

**Table 2:** Descriptive analyses of the continuous independent variables and dependent variables

Dependent variable																							
		N			Prevalence (%)				Mean (SD)				Variance			Median			Minimum		Maximum		
Age 23 D_2+_MFS count		258			69.1				4.75 (6.20)				38.41			3.00			0.00		33.00		
**Continuous independent variables**																							
			**Age 5 to 9**					**Age 9 to 13**							**Age 13 to 17**					**Age 17 to 23**			
**Variables**			**N**			**Mean (SD)**		**N**			**Mean (SD)**				**N**		**Mean (SD)**			**N**			**Mean (SD)**
Combined fluoride intake ^$^			237			0.71 (0.36)		244			0.72 (0.36)				229		0.85 (0.48)			202			1.04 (0.53)
Home water fluoride concentration*			248			0.78 (0.38)		246			0.81 (0.37)				240		0.79 (0.37)			215			0.71 (0.24)
Amount of sugar-free (water-based) beverages intake **			248			1.26 (0.87)		245			1.79 (1.17)				239		2.62 (1.61)			217			4.24 (2.31)
Amount of milk intake **			247			1.67 (0.77)		246			1.50 (0.82)				240		1.56 (1.08)			218			1.11 (0.87)
Amount of 100% juice intake **			249			0.65 (0.45)		245			0.30 (0.33)				241		0.27 (0.31)			219			0.15 (0.33)
Amount of sugar-sweetened beverages intake **			249			0.61 (0.52)		246			1.16 (0.76)				241		1.42 (1.02)			219			1.76 (1.48)
Frequency of sugar-free (water-based) beverages consumption ***			248			1.91 (1.04)		245			1.92 (1.08)				240		2.13 (1.27)			217			3.11 (1.61)
Frequency of milk consumption ***			248			2.13 (0.80)		246			1.66 (0.77)				241		1.42 (0.84)			218			0.95 (0.63)
Frequency of 100% juice consumption ***			249			0.88 (0.57)		245			0.38 (0.41)				241		0.28 (0.32)			219			0.13 (0.24)
Frequency of sugar-sweetened beverages consumption ***			249			0.65 (0.48)		246			0.98 (0.62)				241		0.94 (0.62)			219			1.07 (0.78)
	**Age 9**									**Age 13**								**Age 17**					
	**N**			**%**			**Mean (SD)**			**N**		**%**		**Mean (SD)**				**N**		**%**		**Mean (SD)**	
Previous caries experience (D_2+_MFS)	239			21.8			0.46 (1.08)			247		38.5		1.15 (2.11)				239		62.8		2.94 (4.34)	

All values except age 9, 13, and 17 D_2+_MFS are based on cumulative exposure calculated via AUC using the trapezoidal rule

^$^ milligrams fluoride (mg F) per day; * parts-per-million fluoride (ppm F); ** number of cups (8 oz) per day; ** number of servings per day

As shown in Table 3, D_2+_MFS_23_ count was significantly associated with family income, composite SES and age 9 to 13 cumulative estimates of participants’ brushing frequency (*p* < 0.05). There were significant correlations between D_2+_MFS_23_ count and caries experience at ages 9, 13, and 17, respectively (*r* = 0.28, 0.56, and 0.73, respectively; p-values < 0.001). D_2+_MFS_23_ count was negatively associated with cumulative estimates of frequency of milk intake at ages 5 to 9 and 9 to 13 (*r* = -0.12, -0.13, respectively, *p* < 0.05) and positively associated with cumulative estimates of age 13 to 17 total fluoride intake, age 5 to 9 amount and frequency of sugar-sweetened beverages, and amount and frequency of sugar-sweetened beverages at ages 9 to 13 and 13 to 17 (*r* = 0.14, 0.22, 0.21, 0.28, 0.26, 0.29 and 0.31, respectively, *p* < 0.05).

**Table 3:** Bivariate analyses of the relationships between the independent variables and the dependent variable of age 23 cavitated caries count

Dependent variables: Age 23 D_2+_MFS count *N* = 258							
Test type	Categorical independent variables				Test statistics		*P*-value
Kruskal Wallis chi-square test	Family income				K = 10.23		0.016
	Mother’s level of education				K = 5.96		0.202
	Composite SES				K = 8.76		0.013
Mann-Whitney U test	Sex				W = 7469		0.279
	Age 5–9 cumulative estimate for brushing frequency				W = 6569		0.061
	Age 9–13 cumulative estimate for brushing frequency				W = 6473		0.002
	Age 13–17 cumulative estimates for brushing frequency				W = 4252		0.076
	Age 17–23 cumulative estimates for brushing frequency				W = 3101		0.047
Spearman (Rho) correlation	D_2+_MFS count at 9				*r* = 0.28		< 0.001
	D_2+_MFS count at 13				*r* = 0.56		< 0.001
	D_2+_MFS count at 17				*r* = 0.73		< 0.001
Spearman (Rho) correlation	Continuous independent variables	Age 5 to 9 cumulative estimate	Age 9 to 13 cumulative estimate	Age 13 to 17 cumulative estimate		Age 17 to 23 cumulative estimate	
	Total fluoride intake	*r* = 0.05	*r* = 0.06	*r* = 0.14 *		*r* = 0.06	
	Home fluoride water concentration	*r* = 0.01	*r* = -0.01	*r* = 0.03		*r* = -0.02	
	Amount of sugar-free beverages intake	*r* = -0.10	*r* = -0.02	*r* = -0.02		*r* = 0.06	
	Amount of milk intake	*r* = -0.10	*r* = -0.09	*r* = -0.03		*r* = -0.09	
	Amount of 100% juice intake	*r* = 0.10	*r* = -0.06	*r* = 0.00		*r* = -0.01	
	Amount of sugar-sweetened beverages intake	*r* = 0.22*	*r* = 0.28*	*r* = 0.29*		*r* = 0.09	
	Frequency of sugar-free (water-based) beverages consumption	*r* = -0.12	*r* = -0.09	*r* = -0.10		*r* = -0.03	
	Frequency of milk consumption	*r* = -0.12	*r* = -0.13	*r* = -0.07		*r* = -0.11	
	Frequency of 100% juice consumption	*r* = 0.06	*r* = -0.07	*r* = -0.02		*r* = -0.01	
	Frequency of sugar-sweetened beverages consumption	*r* = 0.21*	*r* = 0.26*	*r* = 0.31*		*r* = 0.09	

r = correlation coefficie

*p < 0.05

### Multivariable model prediction and performance

As shown in Table 4, the best performing model was from the LASSO regression, with a RMSE of 0.70, R^2^ of 0.44, and MAE of 0.48. The GBM and the Negative binomial GLM also performed fairly well, with RMSE scores of 0.74, and 0.76, respectively. The worst performing model was the XGBOOST, with RMSE score of 0.79. More details on the model performance are provided in Appendix V. The lower RMSE and a boxplot showing the comparison of the performance metrics (RMSE, R^2^, and MAE) across all four ML models can be found in Appendix VI. The observed values were found to be calibrated well with the predicted values, as shown in the calibration plot (Appendix VII). After dichotomization from the LASSO model, the classification results (Table 4) showed an accuracy of 83.7%, precision (positive predictive value) of 85.9%, recall (sensitivity) of 93.1%, and ROC AUC of 80.6%.

**Table 4:** Generalization performance of all the predictive models and performance of the LASSO regression model (best performing model) on a binary scale

Model Type (*N* = 258)	Performance metrics			
	RMSE	*R* ^2^		MAE
LASSO regression	0.70	0.44		0.48
Gradient boosting (GBM)	0.74	0.36		0.52
Generalized linear (Negative Binomial) model	0.77	0.31		0.52
Extreme gradient boosting (XGBT)	0.79	0.30		0.55
**Performance metrics of the LASSO model on a binary scale**			**Values (%)**	
Accuracy			83.7%	
Precision			85.9%	
Recall			93.1%	
ROC AUC			80.6%	

*Note* RMSE = root mean square error; R^2^ = coefficient of determination; MAE = Mean absolute error

The assessment of variable importance (Table 5) showed that 4 of the 51 independent variables (age 13 caries count, age 13 caries count, the amount of sugar-sweetened beverages intake from age 9 to 13, and the frequency of sugar-sweetened beverages intake from age 13 to 17) were important in the prediction of and all were positively associated with the cavitated caries outcome count at age 23. The age 17 caries count was the most important variable in the prediction of the D_2+_MFS_23_ count (see Appendix VIII for variable importance plot).

**Table 5:** Variable importance and beta-coefficients from the LASSO regression model

Variables (*N* = 258)	Beta-Coefficients	Relative influence
Age 17 caries experience count	0.546	100
Age 13 caries experience count	0.099	18.18
Amount of sugar-sweetened beverages intake at age 13	0.040	7.27
Frequency of sugar-sweetened beverages intake at age 17	0.033	5.97

Relative influence = the percentage contribution of the predictor variable in the prediction of the outcome variable relative to other variables in the model

## Discussion

Dental caries is a chronic infectious disease with significant public health implications, including in young adults. Our study is one of the first to use machine learning to predict cavitated caries outcomes in young adults from using longitudinally obtained behavioral, and dietary variables.

Our study found a relatively high prevalence of cavitated caries, similar to the findings from the Garcia-Cortes et al. [4] and Jamieson et al. [7] studies conducted within the same age group. However, other studies had much higher mean DMFT/S and percentage prevalence (D_2+_MFS > 0) for this age group compared to our study [5, 6]. These variations might have been due to the variation in the studies’ caries assessment methods, geographic differences and time periods, with caries rates now generally lower overall than in the past.

Exploratory data analysis showed that the D_2+_MFS_23_ count was significantly correlated with family income and composite SES, agreeing with Ismail et al.’s study [3], but contradicting Drachev et al.’s study [5]. Also, the correlations between D_2+_MFS_23_ count and previous caries experience at 9, 13, and 17, found in our study are consistent with the conventional knowledge and findings of other studies [21–23].

Out of all four of the ML models assessed, LASSO regression was the best-performing model, followed by GBM, then GLM (negative binomial), and lastly the XGBOOST model. The LASSO model had the lowest error rate (RMSE and MAE) and highest R-squared compared to the rest of the models. This is contrary to our conventional approach in traditional statistics where count data are usually analyzed using Poisson regression or negative binomial regression models. This clearly demonstrates one of the capabilities of ML to objectively identify models that best fit and explain the variability in the data, rather than relying on statistical assumptions as in regular statistics. Based on the R-squared, only about 44% of the variability in the age 23 caries counts was explained by the variables in the model. A limitation of the use of only R-squared as a performance metric is that it cannot indicate prediction bias in a model (i.e., bias-variance trade-off). It does not tell if the model adequately fits the data or not.

With the discretization and dichotomization of the observed and predicted values of the LASSO model, the model was 84% accurate overall in predicting whether or not a young adult will have caries given their previous caries experience and exposure to dietary, fluoride, and behavioral elements. Our study’s precision (86%) and recall (93%) mean that only 14% were wrongly diagnosed as having had caries experience when they did not, while only 7% of those who had caries experience were misdiagnosed and predicted as having had no caries. There are no other similar studies in children, adolescents, young adults, middle-aged, or older adults with which to compare our findings.

We identified four variables (age 13 caries experience, age 17 caries experience, the amount of sugar-sweetened beverages intake from age 9 to 13, and frequency of sugar-sweetened beverages intake from age 13 to 17) as the most important ones in the prediction of age 23 cavitated caries counts. Age 17 caries experience was the most important predictor of caries counts in young adults, followed by the age 13 caries count, then the amount of sugar-sweetened beverages intake at age 13, and finally, the frequency of sugar-sweetened beverages intake at age 17. This agrees with our hypotheses and conventional knowledge that there are positive associations between caries outcomes and consumption of sugar-sweetened beverages and previous caries experiences. Other variables like total fluoride intake, SES, and brushing frequency which were significant in the bivariate analysis were not selected in the final model. Our finding also suggests that it takes about 5 to 10 years for the teeth to show obvious cavitation following exposure to sugar-sweetened beverages. The policy implication of this finding suggests that it will take about 5 to 10 years to truly observe the effects of preventive oral health interventions such as sugar taxes on caries outcomes at a population level.

The limitations of the study include the moderate sample size, inability to include all possible explanatory variables like genetic variables, and non-generalizability of the findings due to the local nature of the data (mostly non-Hispanic white and higher than average SES Iowans). We attempted to address the issue of limited sample size by using the nested resampling technique with cross-validation. The addition of other variables, like genetic factors, oral bacterial profiles, dental visits, malocclusion, and other systemic diseases might help improve the accuracy and precision of the predictive models.

This study is unique and innovative because it is the first study to use machine learning to predict a cavitated caries experience outcome in young adults using longitudinal obtained fluoride, dietary, and behavioral variables. The longitudinal predictor variables and the use of data from prior years to make predictions add some level of temporality to our study, allowing us to attribute some level of causality to our study findings and prediction. The use of nested resampling with cross-validation helped minimize bias in prediction by ensuring multiple portions of the data were prospectively used in the prediction of the caries outcome. Finally, unlike regular statistical modeling, the choice of an ML model like LASSO regression allowed for the capability of performing dimensionality reduction and feature (variable) selection, as well as assessment of variable collinearity and possible interactions among predictor variables.

## Conclusion

Our ML model generated an accurate, sensitive, and precise model for caries prediction of caries in young adults using longitudinally obtained exposure variables. Our model suggests that continued exposure to a sugary diet for about 5 to 10 years could result in cavitated caries. Our ML algorithm could, in the future, after further development and validation with other diverse population data, be used by dentists and non-dentists as a screening tool to identify the risk of caries in young adults. This will facilitate the translation of caries research into actionable insights that can help improve the quality of life of young adults.

### Electronic supplementary material

Below is the link to the electronic supplementary material.

Supplementary Material 1

## Data Availability

The data that support the findings of this study are available on request from the corresponding author. The data are not publicly available due to privacy or ethical restrictions. We are currently working to share all original Iowa Fluoride Study/Iowa Bone Development Study data later in 2023 through the dbGaP repository under U01- DE028522.
