# Supplementary material for: Predicting dental caries outcomes in young adults using machine learning approach
Source: BMC Oral Health. 2024 May 3;24:529. doi: 10.1186/s12903-024-04294-7 (PMC11069237; doi:10.1186/s12903-024-04294-7)
Supplement: Supplementary file 1 — Supplementary Material 1 [file 12903_2024_4294_MOESM1_ESM.docx]

**APPENDICES**

**Appendix I: Calculation of cumulative exposure variables using the trapezoidal rule for area-under-the-curve estimation**

The cumulative exposure area-under-the-curve (AUC) is a numerical integration of the predictor variables into AUC estimates using the trapezoidal rule. It is a method used for numerical integration or aggregation of data collected at different time points within an observation period into a single variable (Rahman 1990; Weideman 2002). The cumulative exposure area-under-the-curve (AUC) estimates of the independent variables were used to summarize the risk and protective factors for dental caries in this study. The fluoride and beverage consumption AUC estimates were determined by calculating the weighted average of intakes over a set period of observation using the trapezoidal rule for AUC.


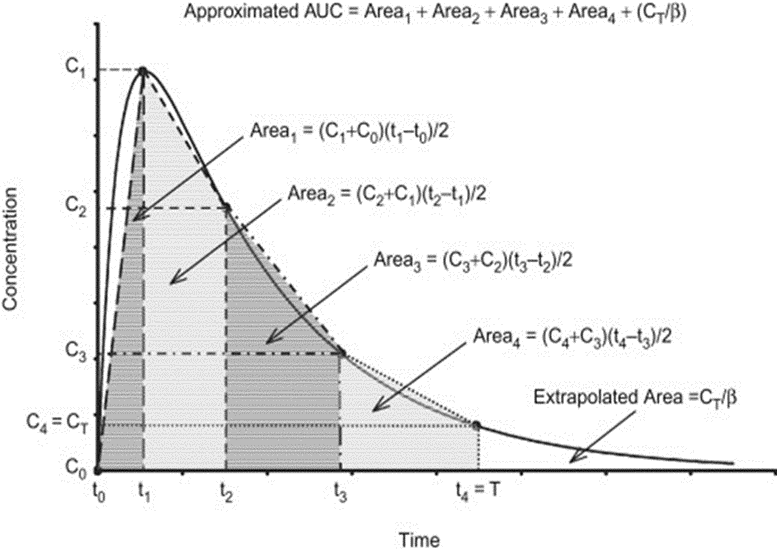
**Eligibility criteria for inclusion of a participant’s data and the calculation of the cumulative exposure of AUC estimate via the trapezoidal rule** were: 1) The participant must have returned at least two questionnaires within the period of interest (e.g., two questionnaires must have been returned between ages 17 and 23) and 2) The subject must have returned at least one questionnaire within one year before or one year after the starting age and another questionnaire within the year before or the year after the ending age (e.g., one questionnaire between ages 16.0 and 18.0 for age 17 and another questionnaire between ages 22.0 and 24.0 for age 23 (Personal communication with Alex Curtis, April 2019)).

The Iowa Fluoride Study data used for this study were questionnaires from ages 9 to 23. Each variable was summarized over four age ranges using a trapezoidal area under the curve. The four age ranges were 5 – 9, 9 – 13, 13 – 17, and 17 – 23 years. The trapezoidal area under the curve for each subject was calculated by first interpolating the first and the last values from the response within the age range of interest. Then, each questionnaire response between the two value points was plotted, with the value of the predictor variable plotted on the y-axis and age plotted on the x-axis. Trapezoids were traced by drawing horizontal lines from each response point down to the x-axis. The area of these trapezoids was summed and divided by the age range (e.g., 6 years for the age 17.0 – 23.0 AUC).

**Appendix II:** **General Iowa Fluoride Study procedures and definitions of variables**

General Iowa Fluoride Study Procedures

This study was a part of the Iowa Fluoride Study – a prospective cohort study that had data collection completed in February 2019. The recruitment of Iowa Fluoride Study participants was done at birth in the post-partum wards of eight Iowa hospitals from March 1992 to February 1995, with follow-up with oral health questionnaires and examinations (Levy et al., 2006). Dental examinations were carried out approximately every four years (except for ages 17 to 23, which was an interval of about 6 years) and oral health questionnaires were distributed approximately every six months (Levy et al., 2006). Some of the factors/variables that were assessed during the process of recruitment were: the age of parents, level of education, and family income (Levy 2001).

Dental Examinations

According to the study protocol, the IFS dental examinations were done by one of the five trained and calibrated dentists using portable dental equipment and halogen lights. After drying the teeth, a DenLite® mirror (Welsh-Allyn Medical Producted, Inc., Skaneatele Falls, NY) was used to enhance lighting and transillumination. No radiographs were used, and examination was based on visualization only; however, gentle explorer probing was used to confirm scoring when in doubt.

At least three out of the five dental examiners performed the dental examinations at each of approximate ages 5, 9, 13, 17, and 23. The dental exams were performed either at the University of Iowa College of Dentistry in Iowa City, IA, or at remote locations for those who could not make it to Iowa City, including Waterloo, IA, and Des Moines, IA. Surface-specific caries was recorded as either sound/arrested (d_0_/D_0_), non-cavitated (d_1_/D_1_) or cavitated (d_2+_/D_2+_) lesions. Teeth with dental sealants were recorded separately and those with restorations were recorded as filled (F). A subject is said to have cavitated dental caries when they have 1 or more D_2+_MFS (or d_2+_mfs) in the oral cavity.

### Risk Factor Assessment Questionnaire

The Iowa Fluoride Study sent out questionnaires to the participants every 3 months to 4 months before age 5 and every 6 months after age 5 to assess their oral hygiene and related behaviors, dietary consumption and exposures to fluoride from water, other beverages, selected foods, fluoride dentifrices, and dietary fluoride supplements (Levy 2001).

1. Oral hygiene-related behaviors

The oral health-related behavior of interest was the frequency of brushing per day which was dichotomized by re-grouping the data. The data collected over the age range of interest (as described above) were averaged to calculate the area-under-the-curve (AUC) estimates for each subject. The AUC estimates for the various oral hygiene-related behaviors were the predictor variables for that period.

1. Exposures to fluoridated water

The main study questionnaire asked the subjects about the main source of their drinking water at home (well vs. Rural Water Association (RWA) vs. city water supply) and the location.^10^ Subjects were asked if they consumed water from home, school, bottled, and other places (where the subject spent at least two full weeks during that questionnaire’s study period). The fluoride exposure from water consumption was calculated based on the average number of ounces of water consumed per day, the duration of that water consumption (during the period with a maximum of 26 weeks), whether the water was filtered, and the type of filtration. The information about water fluoride levels was obtained from the Iowa Department of Public Health for public water supplies, as well as by testing with fluoride-ion electrodes the water samples collected from subjects’ well water or filtration systems. Details are provided in the section on the operational definitions (Levy 2006; Chankanka 2011).

1. Fluoride exposures and intake from toothpaste

The questionnaire asked whether the subject used toothpaste in the previous six months and the brand of the toothpaste they used (Levy et al., 2001). If they used toothpaste, then participants were asked to indicate the amount of toothpaste they usually used during toothbrushing by choosing from seven pictures with different amounts of toothpaste (~0.063mg, 0.125mg, 0.25mg, 0.5 mg, 0.75 mg, 0.875 mg, and 1.0 mg, as well as estimated percentages ingested.

1. Beverage exposure variables (IFS questionnaire)

Information on beverage consumption was obtained by asking the types and brands of beverages they consumed using the IFS questionnaire (Levy 2006; Chankanka 2011). The beverages examined were water, ready-to-drink beverages (tea, coffee, juice and juice drinks, sports drinks, soda pop, coffee, tea, protein drinks, and energy drinks), reconstituted beverages from powder, reconstituted beverages from frozen /liquid concentrate, coffees and tea (brewed or powdered), milk, and, at older ages, wine, beer, and mixed drinks. Prior to the age 9 questionnaire, 100% juice and juice drinks were not separated and the “sugar-free beverages” category only contained water. After age 9, juice drinks were added to the sugar-sweetened beverage category, and beverages such as diet soda and powdered beverages such as Crystal Light^TM^ were included in the “sugar-free beverages” category. The beverage exposures first were assessed on a dichotomous scale (whether or not the individual consumed the beverage). Among individuals who had that beverage, the number of servings per week and the amount per serving (in ounces) were then recorded and used to calculate the overall intakes of the beverages in servings and ounces per week. The details on each of the variables are provided in the next section on operational definitions.

**Operational definitions**

Dental caries

The main caries outcome was defined as a person-level D_2+_MFS count. The D_2+_MFS count was calculated as the sum of decayed (D_2+_/cavitated lesion), missing (M) and filled (F) surfaces. Dental caries was analyzed as a count variable for univariate, bivariate, and multivariable analyses. However, the dental caries outcome also was collapsed into two categories (D_2+_MFS = 0 vs. D_2+_MFS > 0) to calculate the percentage prevalence.

Sex

The sex of the participants was either male or female, as specified in the recruitment questionnaire.

Mother’s level of education

This was defined as the highest level of education achieved by the female head of household in 2007 (some high school, high school, some college, 2-year college degree, 4-year college degree, advanced degree, no female head of household). This was defined as an ordinal variable.

Family income level

Based on the 2007 demographic questionnaire, the annual family income level was defined as less than $20,000, $20,000 - $39,999, $40,000 - $59,999, $60,000 - $79,999, and greater than or equal to $80,000. Unlike the Ahuja et al. (2013) study that defined family income as a categorical variable, the annual family income was defined as an ordinal variable for both bivariate and multivariable analysis.

Daily brushing frequency category

This was defined as the number of toothbrushings per day which was calculated by using the area-under-the-curve (AUC) estimate. The area-under-the-curve (AUC) estimate is the weighted average of the responses obtained from the IFS questionnaire collected over a specified period. For example, age 17.0 to 23.0 IFS questionnaires were used to calculate the age 17 to 23 AUC estimates of the daily toothbrushing frequency category. The unit of measurement was the number of brushing events/day. The AUC estimates of this variable which was continuous data were dichotomized for easier interpretability. AUC estimates less than 1.5 were categorized as 1 (that is, subjects brush once or less per day) while AUC estimates 1.5 and above were categorized as 2 (that is, subjects brush 2 or more per day).

Composite socioeconomic status

This variable is a 3-category variable. The high, middle, and low categories are defined based on the combination of two variables, mother’s educational level and family income. The formula for categorization was: less than graduate/professional school + <$40,000 = Low SES; less than graduate/professional school + $40,000-$79,999 = Middle SES; less than graduate/professional school + $80,000 or more = High SES; graduate/professional school + <$40,000 = middle SES; graduate/professional school + $40,000-$79,999 = High SES; and graduate/professional school + $80,000 or more = High SES.

Composite water fluoride level

This was defined as the weighted average level of fluoride in each of the subjects’ major water sources at each assessment point. This was reported using the area-under-the-curve (AUC) estimate which is the weighted average of the responses obtained from the IFS questionnaire collected over a specified period. For example, responses from age 17.0 to 23.0 IFS questionnaires were used to calculate the age 17 to 23 AUC estimate of daily fluoride intake from subjects’ main sources. The variable was considered as a continuous variable in parts per million (ppm) of fluoride.

Daily fluoride intake from home water

This was defined as the sum of the weighted average amount of fluoride in each of the subjects’ home water sources. The variable was a continuous variable in milligrams of fluoride (mgF) /day. This was reported using the area-under-the-curve (AUC) estimate which is the weighted average of the responses obtained from the IFS questionnaire collected over a specified period. For example, responses from age 17.0 to 23.0 IFS questionnaires were used to calculate the age 17 to 23 AUC estimate of daily fluoride intake from home water.

Daily fluoride intake from combined sources

This was defined as the total daily intake of fluoride from all sources (dentifrice, water, beverages, and some foods with added water (e.g., soup, rice, ramen noodles, etc.). This was reported as the area-under-the-curve (AUC) estimate which is the weighted average of the responses obtained from the IFS questionnaires collected over a specified period. For example, responses from age 17.0 to 23.0 IFS questionnaires were used to calculate the age 17 to 23 AUC estimate of daily fluoride intake from combined sources. The variable was considered as a continuous variable in mgF per day.

Frequency of sugar-sweetened beverage consumption

This was defined as the number of times a subject consumed a beverage with added sugar per day. This was reported as the area-under-the-curve (AUC) estimate which is the weighted average of the responses obtained from the IFS questionnaire collected over a specified period. For example, responses from age 17.0 to 23.0 IFS questionnaires were used to calculate the age 17 to 23 AUC estimate of the frequency of the sugared beverage consumption. It was considered as the number of servings per day (continuous variable).

Daily intake of sugar-sweetened beverage

This was defined as the amount/quantity of beverage with added sugar consumed by the subject per day. This was reported as the area-under-the-curve (AUC) estimate which is the weighted average of the responses obtained from the IFS questionnaire collected over a specified period. For example, responses from age 17.0 to 23.0 IFS questionnaires were used to calculate the age 17 to 23 AUC estimate of the daily intake of sugar-sweetened beverages. It was considered as the amount of sugar-sweetened beverages intake in cups (8 oz) per day (continuous variable).

Frequency of 100% juice consumption

This was defined as the number of times a subject consumed 100% juice containing natural sugar and no added sugar per serving per day. This was reported as the area-under-the-curve (AUC) estimate which is the weighted average of the responses obtained from the IFS questionnaire collected over a specified period. For example, responses from age 17.0 to 23.0 IFS questionnaires were used to calculate the age 17 to 23 AUC estimate of the frequency of 100% juice consumption It was defined as the number of servings per day (continuous variable).

Daily intake of 100% juice

This was defined as the amount/quantity of 100% juice containing natural sugar and no added sugar consumed by the subject per day. This was reported as the area-under-the-curve (AUC) estimate which is the weighted average of the responses obtained from the IFS questionnaire collected over a specified period. For example, responses from age 17.0 to 23.0 IFS questionnaires were used to calculate the age 17 to 23 AUC estimate of the daily intake of 100% juice. It was considered as the amount of 100% juice intake in cups (8 oz) per day (continuous variable).

Frequency of sugar-free beverage consumption (no sugar added)

The frequency of sugar-free beverage consumption was defined as the number of times a subject consumed a beverage with no added sugar (artificial or natural) and/or plain water per day. This was reported as the area-under-the-curve (AUC) estimate which is the weighted average of the responses obtained from the IFS questionnaire collected over a specified period. For example, responses from age 17.0 to 23.0 IFS questionnaires were used to calculate the age 17 to 23 AUC estimate of the frequency of sugar-free beverage consumption. It was considered as the number of servings per day (continuous variable).

Daily intake of sugar-free beverage (no sugar added)

This was defined as the amount/quantity of beverage with no added sugar (artificial or natural) and/or plain water consumed by the subject per day. This was reported as the area-under-the-curve (AUC) estimate which is the weighted average of the responses obtained from the IFS questionnaire collected over a specified period. For example, responses from age 17.0 to 23.0 IFS questionnaires were used to calculate the age 17 to 23 AUC estimate of the daily intake of sugar-free beverages. It was considered as the amount of sugar-free beverages intake in cups (8 oz) per day (continuous variable).

Frequency of milk consumption

This was defined as the number of times a subject consumed milk per serving per day. This was reported as the area-under-the-curve (AUC) estimate which is the weighted average of the responses obtained from the IFS questionnaire collected over a specified period. For example, responses from age 17.0 to 23.0 IFS questionnaires were used to calculate the age 17 to 23 AUC estimate of the frequency of milk consumption. It was defined as the number of servings per day (continuous variable).

Daily intake of milk

This was defined as the amount/quantity of milk consumed by the subject per day. This was reported as the area-under-the-curve (AUC) estimate which is the weighted average of the responses obtained from the IFS questionnaire collected over a specified period. For example, responses from age 17.0 to 23.0 IFS questionnaires were used to calculate the age 17 to 23 AUC estimate of the daily intake of milk. It was considered as the amount of milk intake in cups (8 oz) per day (continuous variable).

Summary of definition of variables

1. Daily toothbrushing frequency category was defined as the number of toothbrushings per day. AUC estimates less than 1.5 were categorized as 1 (that is, subjects brush once or less per day) while AUC estimates 1.5 and above were categorized as 2 (that is, subjects brush 2 or more per day).
2. Daily fluoride intake from combined sources: AUC estimate of the total daily intake of fluoride from all sources (dentifrice, water, beverages, and some foods with added water (e.g., soup, rice, noodles, etc.)), fluoride dentifrice and dietary fluoride supplements, in mg F per day.
3. Concentration of fluoride in-home water: AUC estimate of the weighted average amount of fluoride in subject’s home water sources in parts per million fluoride (ppm F) at different times.
4. Daily intake of sugar-free beverages (no sugar added): AUC estimate of the amount/quantity of beverage with no added sugar (artificial or natural) and/or plain water consumed by the subject per day, i.e., the amount of sugar-free beverages intake in cups (8 oz) per day.
5. Daily intake of milk: AUC estimate of the amount of milk intake in cups (8 oz) per day.
6. Daily intake of 100% juice: AUC estimate of the amount/quantity of 100% juice containing natural sugar and no added sugar in cups (8 oz) per day.
7. Daily intake of sugar-sweetened beverages: AUC estimate of the amount/quantity of beverages with added sugar consumed in cups (8 oz) per day.
8. Frequency of sugar-free beverage consumption: AUC estimate of the number of times a subject consumed a beverage with no added sugar (artificial or natural) and/or plain water per day.
9. Frequency of milk intake: AUC estimate of the number of times a subject drank milk per day.
10. Frequency of 100% juice consumption: AUC estimate of the number of times a subject consumed 100% juice containing natural sugar and no added sugar per day.
11. Frequency of sugar-sweetened beverages consumption: AUC estimate of the number of daily servings of sugar-sweetened beverages per day.
12. Age 9, 13, and 17 dental caries experience measures were defined as the person-level D_2+_MFS counts at each of these exams, defined as the sum of the numbers of decayed (D_2+_/cavitated lesion), missing (M), and filled (F) surfaces.
13. Sex was defined as male or female.
14. Family income level was defined in five categories based on the data from a questionnaire in 2007 as less than $40,000, $40,000 to $59,999, $60,000 to $79,999, and >=$80,000.
15. Mother’s level of education was defined in five categories based on the data from a questionnaire in 2007 as high school or lower, some college, 2-year college degree, 4-year college degree, and advanced degree.
16. Composite SES: This variable is a 3-category variable (low, middle and high SES). They are defined based on the combination of two variables (mother’s educational level and family income. The formula for categorization was: less than graduate/professional school + <$40,000 = low SES; less than graduate/professional school + $40,000-$79,999 = middle SES; less than graduate/professional school + $80,000 or more = high SES; graduate/professional school + <$40,000 = middle SES; graduate/professional school + $40,000-$79,999 = high SES; and graduate/professional school + $80,000 or more = high SES.

**Appendix III: Definitions of the machine learning models used**

Least Absolute Shrinkage and Selection Operator (LASSO) regression

LASSO regression is a type of linear regression that uses a shrinkage method in reducing the complexity of a model (Tibshirani 1996). It can also be extended to other forms of regression such as logistic and Poisson regression. LASSO regression performs L1 regularization, which minimizes the loss function by adding a penalty equal to the absolute value of the magnitude of coefficients to the loss function of linear regression (sum of squares) (Tibshirani 1996; James 2013). This type of regularization can result in sparse models with fewer variables, causing some coefficients to become zero and “eliminated” from the model. It is well-suited for models with high levels of multi-collinearity and for data where the number of variables is greater than the number of observations (Tibshirani 1996; James 2013).

Gradient Boosting Model (GBM) and Extreme Gradient Boosting Model (XGBOOST)

Gradient boosting modeling is a simple, elegant, and highly adaptable algorithm capable of doing both classification and regression (Greenwell 2019).^3^ The primary idea behind the gradient boosting algorithm is to find the best model by minimization of the loss function. The advantages of the gradient boosting model are the ability to perform variable selection based on variable importance, perform both classification (analysis of categorical outcomes) and regression, and provide a very competitive model performance compared to most machine learning models. The disadvantages are that it is computationally expensive and the interpretation can be complicated due to the iterative processes involved in the computation (Greenwell 2019; Friedman 2001).

Extreme gradient boosting is a modification of gradient boosting modeling. The algorithm follows the same principle as for the gradient boosting model, but with more hyperparameter tuning functions, is computationally faster, and applies regularization to minimize model overfitting (Chen 2016).

**Appendix IV: Descriptive statistics for the continuous independent variables and dependent variable**

|  | **N** | **Mean** | **Standard deviation** | **Variance** | **Median** | **Minimum** | **Maximum** |
| --- | --- | --- | --- | --- | --- | --- | --- |
| Age 23 D_2+_MFS count |  | 4.75 | 6.20 | 38.41 | 3.00 | 0.00 | 33.00 |
| **Age 5 to 9 cumulative exposure AUC estimate variables** |  | **Mean** | **Standard deviation** | **Variance** | **Median** | **Minimum** | **Maximum** |
| Combined fluoride intake ^$^ | 237 | 0.71 | 0.36 | 1.31 | 0.63 | 0.13 | 2.09 |
| Home water fluoride concentration* | 248 | 0.78 | 0.38 | 1.45 | 0.95 | 0.04 | 2.26 |
| **Amount** of sugar-free (water-based) beverages intake ** | 248 | 1.26 | 0.87 | 0.76 | 1.06 | 0.10 | 5.68 |
| **Amount** of milk intake ** | 247 | 1.67 | 0.77 | 0.60 | 1.61 | 0.0 | 5.58 |
| **Amount** of 100% juice intake ** | 249 | 0.65 | 0.45 | 0.21 | 0.54 | 0.0 | 1.65 |
| **Amount** of sugar-sweetened beverages intake ** | 249 | 0.61 | 0.52 | 0.27 | 0.43 | 0.03 | 9.84 |
| **Frequency** of sugar-free (water-based) beverages consumption *** | 248 | 1.91 | 1.04 | 1.08 | 1.73 | 0.60 | 9.65 |
| **Frequency** of milk consumption *** | 248 | 2.13 | 0.80 | 0.64 | 2.14 | 0.00 | 3.35 |
| **Frequency** of 100% juice consumption *** | 249 | 0.88 | 0.57 | 0.32 | 0.79 | 0.00 | 1.63 |
| **Frequency** of sugar-sweetened beverages consumption *** | 249 | 0.65 | 0.48 | 0.23 | 0.51 | 0.02 | 5.13 |
| **Age 9 to 13 cumulative exposure AUC estimate variables** |  |  |  |  |  |  |  |
| Combined fluoride intake ^$^ | 244 | 0.72 | 0.36 | 1.31 | 0.64 | 0.14 | 2.10 |
| Home water fluoride concentration* | 246 | 0.81 | 0.37 | 1.37 | 0.97 | 0.03 | 2.15 |
| **Amount** of sugar-free (water-based) beverages intake ** | 245 | 1.79 | 1.17 | 1.38 | 1.52 | 0.26 | 7.42 |
| **Amount** of milk intake ** | 246 | 1.50 | 0.82 | 0.67 | 1.48 | 0.00 | 5.15 |
| **Amount** of 100% juice intake ** | 245 | 0.30 | 0.33 | 0.11 | 0.22 | 0.00 | 2.11 |
| **Amount** of sugar-sweetened beverages intake ** | 246 | 1.16 | 0.76 | 0.58 | 0.96 | 0.00 | 4.72 |
| **Frequency** of sugar-free (water-based) beverages consumption *** | 245 | 1.92 | 1.08 | 1.17 | 1.69 | 0.23 | 8.75 |
| **Frequency** of milk consumption *** | 246 | 1.66 | 0.77 | 0.59 | 1.65 | 0.00 | 3.71 |
| **Frequency** of 100% juice consumption *** | 245 | 0.38 | 0.41 | 0.17 | 0.27 | 0.00 | 2.72 |
| **Frequency** of sugar-sweetened beverages consumption *** | 246 | 0.98 | 0.62 | 0.39 | 0.83 | 0.00 | 3.80 |
| **Age 13 to 17 cumulative exposure AUC estimate variables** |  |  |  |  |  |  |  |
| Combined fluoride intake ^$^ | 229 | 0.85 | 0.48 | 2.31 | 0.74 | 0.17 | 2.68 |
| Home water fluoride concentration* | 240 | 0.79 | 0.37 | 1.37 | 0.92 | 0.06 | 2.24 |
| **Amount** of sugar-free (water-based) beverages intake ** | 239 | 2.62 | 1.61 | 2.58 | 2.22 | 0.04 | 8.77 |
| **Amount** of milk intake ** | 240 | 1.56 | 1.08 | 1.16 | 1.33 | 0.00 | 6.57 |
| **Amount** of 100% juice intake ** | 241 | 0.27 | 0.31 | 0.10 | 0.15 | 0.00 | 1.32 |
| **Amount** of sugar-sweetened beverages intake ** | 241 | 1.42 | 1.02 | 1.04 | 1.21 | 0.00 | 5.88 |
| **Frequency** of sugar-free (water-based) beverages consumption *** | 240 | 2.13 | 1.27 | 1.61 | 1.77 | 0.04 | 7.99 |
| **Frequency** of milk consumption *** | 241 | 1.42 | 0.84 | 0.70 | 1.29 | 0.00 | 4.09 |
| **Frequency** of 100% juice consumption *** | 241 | 0.28 | 0.32 | 0.10 | 0.16 | 0.00 | 1.34 |
| **Frequency** of sugar-sweetened beverages consumption *** | 241 | 0.94 | 0.62 | 0.38 | 0.77 | 0.00 | 3.87 |
| **Age 17 to 23 cumulative exposure AUC estimate variables** |  |  |  |  |  |  |  |
| Combined fluoride intake ^$^ | 202 | 1.04 | 0.53 | 2.84 | 0.94 | 0.22 | 3.35 |
| Home water fluoride concentration* | 215 | 0.71 | 0.24 | 0.56 | 0.69 | 0.11 | 1.78 |
| **Amount** of sugar-free (water-based) beverages intake ** | 217 | 4.24 | 2.31 | 5.32 | 3.80 | 0.59 | 14.71 |
| **Amount** of milk intake ** | 218 | 1.11 | 0.87 | 0.76 | 0.92 | 0.00 | 4.63 |
| **Amount** of 100% juice intake ** | 219 | 0.15 | 0.33 | 0.11 | 0.04 | 0.00 | 3.91 |
| **Amount** of sugar-sweetened beverages intake ** | 219 | 1.76 | 1.48 | 2.19 | 1.29 | 0.03 | 9.84 |
| **Frequency** of sugar-free (water-based) beverages consumption *** | 217 | 3.11 | 1.61 | 2.58 | 2.83 | 0.60 | 9.65 |
| **Frequency** of milk consumption *** | 218 | 0.95 | 0.63 | 0.40 | 0.87 | 0.00 | 3.35 |
| **Frequency** of 100% juice consumption *** | 219 | 0.13 | 0.24 | 0.06 | 0.04 | 0.00 | 1.76 |
| **Frequency** of sugar-sweetened beverages consumption *** | 219 | 1.07 | 0.78 | 0.62 | 0.85 | 0.02 | 4.82 |
| Previous caries experience **(Age 9 D_2+_MFS**) | 239 | 0.46 | 1.08 | 1.17 | 0.00 | 0.00 | 6.00 |
| Previous caries experience **(Age 13 D_2+_MFS**) | 247 | 1.15 | 2.11 | 4.46 | 0.00 | 0.00 | 11.00 |
| Previous caries experience **(Age 17 D_2+_MFS**) | 239 | 2.93 | 4.33 | 18.82 | 2.00 | 0.00 | 37.00 |
| **Footnotes:**  ^$^ milligrams fluoride (mg F) per day; * parts-per-million fluoride (ppm F); ** number of cups (8 oz) per day; *** number of servings per day  All values except age 9, 13, and 17 D_2+_MFS are based on cumulative exposure AUC estimates | | | | | | | |

**Appendix V: Generalization performance of the predictive models** (N = 258) (GLM family = Negative binomial)

| Metrics | Models assessed | Statistics | | | | |
| --- | --- | --- | --- | --- | --- | --- |
|  |  | Mean | Median | SD | Minimum | Maximum |
| RMSE | GLM | 0.7698 | 0.7476 | 0.1701 | 0.5678 | 1.0557 |
|  | GBM | 0.7437 | 0.7196 | 0.1449 | 0.5445 | 0.9826 |
|  | LASSO | 0.7001 | 0.6368 | 0.2055 | 0.5027 | 1.0476 |
|  | XGBT | 0.7910 | 0.7561 | 0.1592 | 0.6393 | 1.1438 |
| R^2^ |  | Mean | Median | SD | Minimum | Maximum |
|  | GLM | 0.3136 | 0.3518 | 0.2980 | -0.1086 | 0.7323 |
|  | GBM | 0.3603 | 0.4549 | 0.2955 | -0.2264 | 0.6647 |
|  | LASSO | 0.4440 | 0.4105 | 0.2357 | 0.0907 | 0.7849 |
|  | XGBT | 0.2951 | 0.3301 | 0.2537 | -0.1288 | 0.6050 |
| MAE |  | Mean | Median | SD | Minimum | Maximum |
|  | GLM | 0.5164 | 0.5037 | 0.1109 | 0.3885 | 0.7268 |
|  | GBM | 0.5249 | 0.4956 | 0.0981 | 0.4358 | 0.7270 |
|  | LASSO | 0.4817 | 0.4566 | 0.1169 | 0.3683 | 0.7180 |
|  | XGBT | 0.5497 | 0.5084 | 0.1054 | 0.4296 | 0.7689 |
| RMSE = Root mean square error; R^2^ = Coefficient of determination; MAE = Mean Average error  LASSO = Least Absolute Shrinkage and Selection Operator (LASSO) regression,  GLM = Generalized Linear Model (negative binomial regression)  GBM = Gradient boosting modeling  XGBOOST = Extreme gradient boosting modeling | | | | | | |

**Appendix VI: Boxplots showing generalization performance of the predictive models**

The dots within the box are the means, the dots outside the box are outliers, the bold vertical line is the median, the left and right vertical lines are the first and 3^rd^ quartile, respectively, and the horizontal line is the range of values. (N =258)


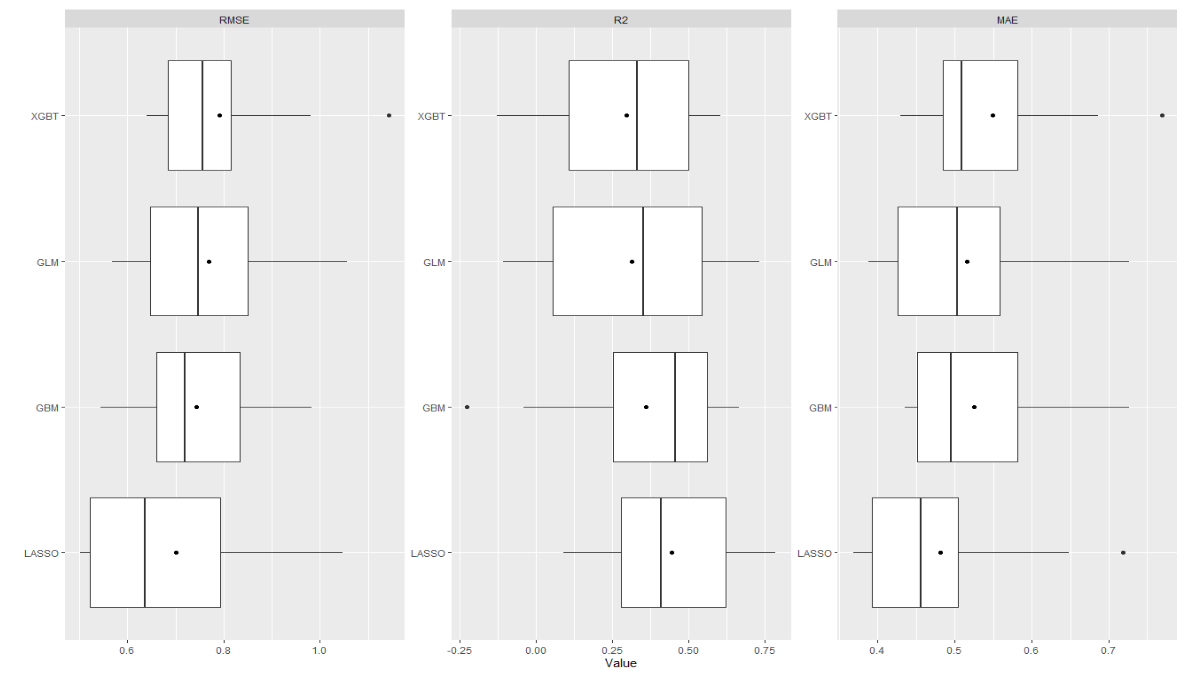


**Appendix VII: Calibration plot of the LASSO model showing the relationship between the observed values and the predicted (fitted) values**. (N = 258)


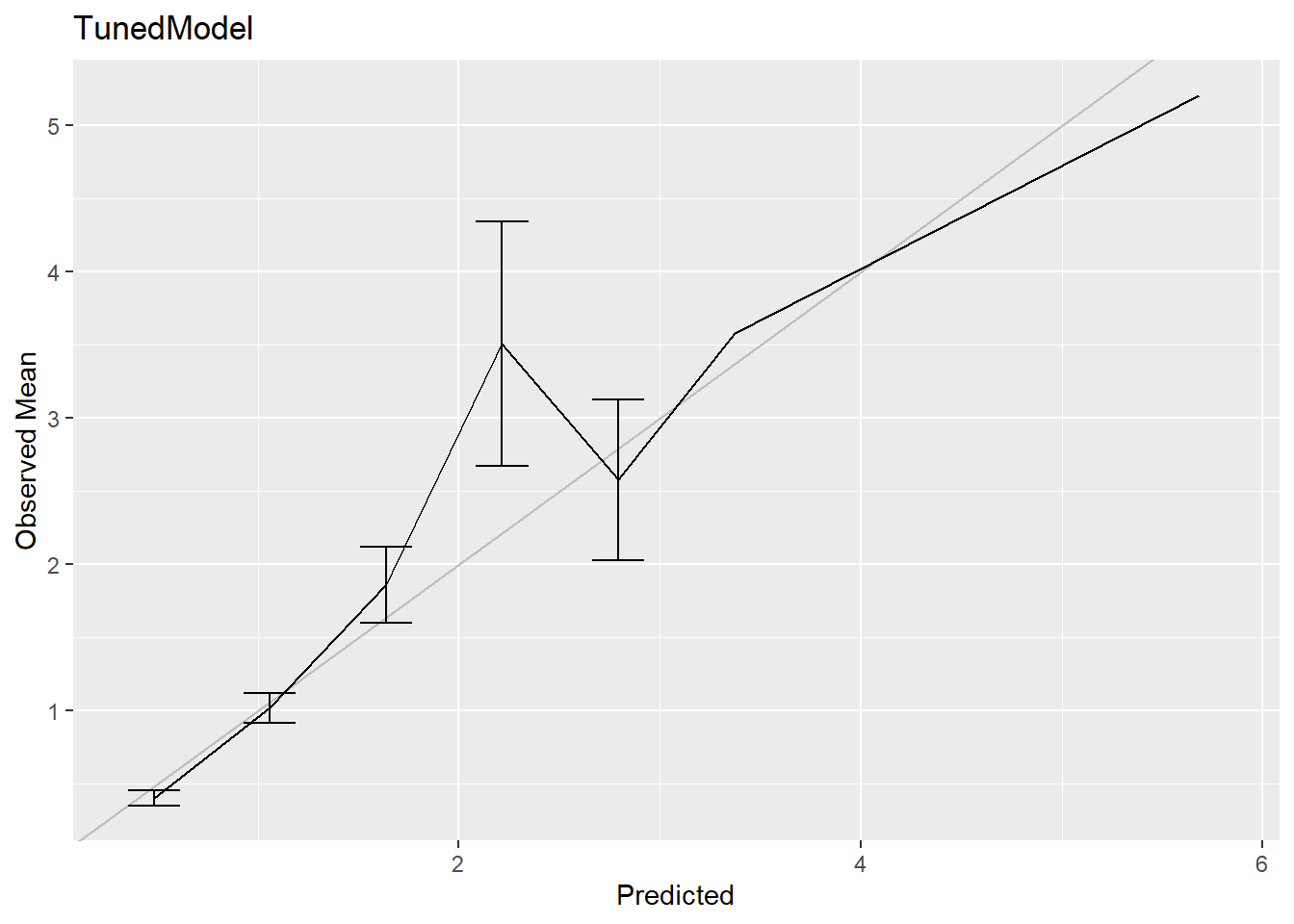


**Note:** The gray line represents the perfect line of fit between the observed and predicted mean of a perfect model; the black line represents the line of fit between the observed and predicted mean of our prediction model (LASSO model))

**Appendix VIII:** **Histogram showing the variable importance plot for the LASSO regression**

**
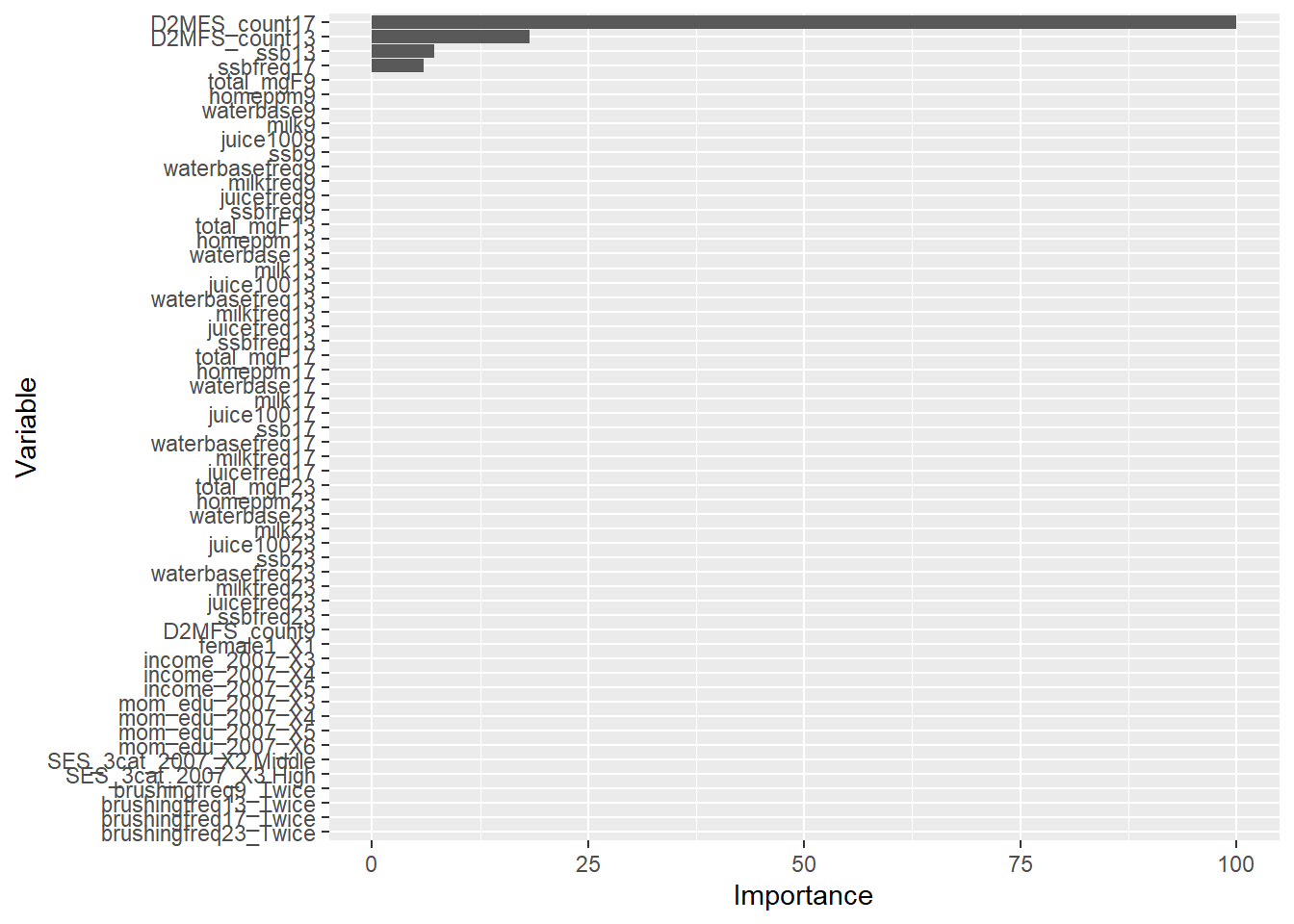
**(N = 258).

**Note:** The plot also represents the results of the feature selection obtained by regularization. The first four histogram bars represent the most influential variables, while the rest without bars represent variables whose coefficients have been shrunken to zero via regularization.

**Appendix IX:** **Machine learning pipeline**


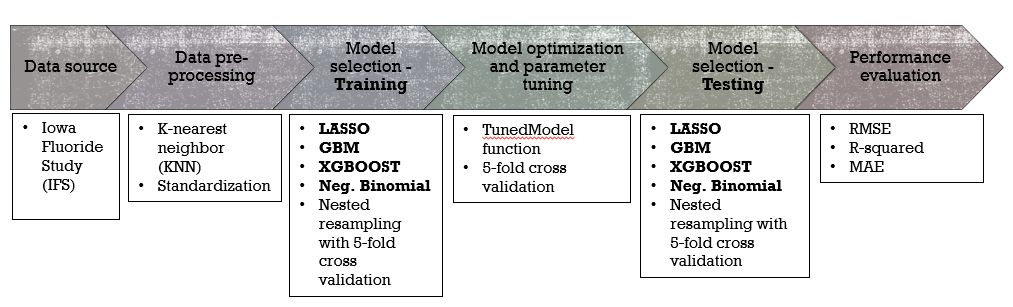


The supervised machine learning modelling was performed using mainly the “recipes” and “MachineShop” packages in R. First, the data were extracted from the existing IFS database and exported to an excel spreadsheet. All data preprocessing was done using the recipes package by standardizing the data and imputation of the missing values using the K-nearest neighbor (KNN) imputation technique. The model selection was performed by training and comparing 4 models - Lasso regression, negative binomial regression, gradient boosting machines (gbm) and extreme gradient boosting machines (Xgboost) using nested resampling technique under 5-fold cross validation. These models were then optimized by tuning them using the TunedModel function in MachineShop package and the tuning parameters were chosen using the cross-validation technique. The models’ performances then were tested with a fresh dataset from the resampled data and the generalization performance was assessed based on the RMSE, R-squared and MAE.

For easier interpretation, the observed and predicted values from the best performing model were discretized, dichotomized and assessed based on more interpretable performance metrics - Accuracy, precision, ROC AUC.

**Appendix X:** **Machine Learning Code** (The code and the dataset can be found at <https://github.com/Drbuxie/Predicting-caries-outcome-in-young-adults> )

################### Loading packages #############################

```{r packages}

#install.packages("PerformanceAnalytics")

#install.packages("haven")

#install.packages("MachineShop")

#install.packages("tidyverse")

#install.packages("doParallel")

#install.packages("magrittr")

#install.packages("recipes")

#install.packages("psych")

#install.packages("gbm")

#install.packages("imputeTS")

#install.packages("nnet")

#install.packages("MASS")

#install.packages("glmnet")

#install.packages("corrplot")

library(corrplot)

library(haven)

library(readr)

library(dplyr)

library(psych)

library(tableone)

require(MASS)

require(knitr)

library(MASS)

library(MachineShop)

library(nnet)

library(stats)

suppressPackageStartupMessages(library(doParallel))

suppressPackageStartupMessages(library(recipes))

library(magrittr)

library(gbm)

## Allocate cores for parallel processing

registerDoParallel(cores = 6)

```

```{r}

##importing complete DMFS dataset

setwd("/Users/damia/OneDrive/Documents/PhD/Datasets")

D2EXP<- read.csv("/Users/damia/OneDrive/Documents/PhD/Datasets/Codes for paper 1, 2, 3/D2EXP.csv")

head(D2EXP)

```

##################### Exploratory data analyses ###########################

```{r}

############# Univariate analysis ###########

#D2EXP %>% glimpse()

summary(D2EXP[,1:9])

stat.desc (D2EXP[,10:52], basic = FALSE)

stat.desc (D2EXP[,53])

stat.desc (D2EXP[,10:53])

#####Bivariate analysis##############

wilcox.test(D2MFS_count23 ~ female1, mu=0, alt= "two.sided", paired= F, conf.int = T, conf.level = 0.95, exact=F, correct=T, data = D2EXP)

wilcox.test(D2MFS_count23 ~ brushingfreq9, mu=0, alt= "two.sided", paired= F, conf.int = T, conf.level = 0.95, exact=F, correct=T, data = D2EXP)

wilcox.test(D2MFS_count23 ~ brushingfreq13, mu=0, alt= "two.sided", paired= F, conf.int = T, conf.level = 0.95, exact=F, correct=T, data = D2EXP)

wilcox.test(D2MFS_count23 ~ brushingfreq17, mu=0, alt= "two.sided", paired= F, conf.int = T, conf.level = 0.95, exact=F, correct=T, data =D2EXP)

wilcox.test(D2MFS_count23 ~ brushingfreq23, mu=0, alt= "two.sided", paired= F, conf.int = T, conf.level = 0.95, exact=F, correct=T, data = D2EXP)

kruskal.test(D2MFS_count23 ~ income_2007, data = D2EXP)

kruskal.test(D2MFS_count23 ~ mom_edu_2007, data = D2EXP)

kruskal.test(D2MFS_count23 ~ SES_3cat_2007, data = D2EXP)

```

```{r,fig.height=10,fig.width=13,options(max.print=1000000) }

############ Bivariate analysis ##############

####### Numerical independent variables: Correlation tests #############

##create dataframe of numerical variables##

BV_cont<-D2EXP[c("total_mgF9","homeppm9","waterbase9","milk9","juice1009","ssb9","waterbasefreq9","milkfreq9","juicefreq9","ssbfreq9",

"total_mgF13","homeppm13","waterbase13","milk13","juice10013","ssb13","waterbasefreq13","milkfreq13","juicefreq13","ssbfreq13",

"total_mgF17","homeppm17","waterbase17","milk17","juice10017","ssb17","waterbasefreq17","milkfreq17","juicefreq17","ssbfreq17",

"total_mgF23","homeppm23","waterbase23","milk23","juice10023","ssb23","waterbasefreq23","milkfreq23","juicefreq23","ssbfreq23",

"D2MFS_count9","D2MFS_count13","D2MFS_count17","D2MFS_count23")]

##perform correlation test using the new dataframe##

corr.test(BV_cont[,unlist(lapply(BV_cont, is.numeric))])

corrplot(cor(BV_cont), method="circle")

chart.Correlation(BV_cont[1:44])

```

############ Data Preprocessing using Recipes package #######################

```{r}

recipe1 <- recipe(D2MFS_count23 ~ ., data = D2EXP) %>%

step_rm(id)%>% ##Removing variable id

step_nzv(all_predictors()) %>% ##Potentially remove variables that are highly sparse and unbalanced

step_normalize(all_numeric(), -all_outcomes()) %>% ##Normalize all numeric data

step_scale(all_numeric())%>% ##Scaling all numeric variables

#step_dummy(all_nominal(),-all_outcomes())%>%

#step_interact(terms = ~ .)%>%

role_case(stratum = D2MFS_count23)

juice(prep(recipe1))

##imputation

recipe2 <- recipe1 %>%

step_impute_knn(all_predictors())%>% ##Imputation using K-nearest neighbor

step_dummy(all_nominal(), -all_outcomes()) ##Create dummy variables from nomial variables

juice(prep(recipe2))

```

############ Predictive modeling #######################

```{r}

## Resampling Control

cvc <- CVControl(folds = 10, repeats = 1, seed = 808)

```

```{r}

#################### Model 1 - Negative Binomial regression #####################

model<- TunedModel(GLMStepAICModel(family = "negbin"))

glms_fit <- fit(recipe2, model = model)

glms_res <- resample(recipe2, model = model, control = cvc)

summary(glms_res)

```

```{r}

## Negative binomial regression - parameter tuning and plots

N_tuned_model <- as.MLModel(glms_fit)

print(N_tuned_model, n = Inf)

#mean(glms_res$Observed)

GLM_VImp = varimp(glms_fit)

GLM_calib = calibration(glms_res)

#GLM_Pdep = dependence(glms_fit)

#plot(N_tuned_model, type = "line")

plot(GLM_VImp)

#plot(GLM_Pdep)

plot(GLM_calib, se=TRUE)

```

```{r}

#################### Model 2 - Lasso regression #####################

model <- TunedModel(GLMNetModel, grid = 10, fixed = list(alpha = 1))

lasso_fit <- fit(recipe2, model = model)

lasso_res <- resample(recipe2, model = model, control = cvc)

summary(lasso_res)

summary(lasso_fit)

```

```{r}

##Lasso regression - parameter tuning and plots

L_tuned_model <- as.MLModel(lasso_fit)

print(L_tuned_model, n = Inf)

#mean(lasso_res$Observed)

Lasso_VImp = varimp(lasso_fit)

Lasso_calib = calibration(lasso_res)

#Lasso_Pdep = dependence(lasso_fit,select = c("waterbasefreq9","ssb23","ssb13", "ssbfreq17", "D2MFS_count13", "D2MFS_count17", "mom_edu_2007", "brushingfreq13"))

plot(L_tuned_model, type = "line")

Lasso_VImp

plot(Lasso_VImp)

#plot(Lasso_Pdep)

plot(Lasso_calib, se=TRUE)

```

```{r}

############### Model 3 - gradient boosting machines (GBM)##############

model <- TunedModel(GBMModel(distribution = "poisson"))

GBM_fit <- fit(recipe2, model = model)

GBM_res <- resample(recipe2, model = model, control = cvc)

summary(GBM_res)

summary(GBM_fit)

```

```{r}

##GBM - parameter tuning and plots

GB_tuned_model <- as.MLModel(GBM_fit)

print(GB_tuned_model, n = Inf)

#mean(GBM_res$Observed)

GBM_VImp = varimp(GBM_fit)

GBM_calib = calibration(GBM_res)

#GBM_Pdep = dependence(GBM_fit)

plot(GB_tuned_model, type = "line")

plot(GBM_VImp)

#plot(GBM_Pdep)

plot(GBM_calib, se=TRUE)

```

```{r}

############ Model 4 - Extreme gradient boosting model (XGBOOST) ############

model <- TunedModel(

XGBTreeModel(

nrounds= 10,

lambda =1,

alpha= 0,

max_depth = 3,

verbose = 2,

objective = "count:poisson"

)

)

XGT_fit <- fit(recipe2, model = model)

XGT_res <- resample(recipe2, model = model, control = cvc)

summary(XGT_res)

summary(XGT_fit)

```

```{r}

## XGBOOST - parameter tuning and plots

XG_tuned_model <- as.MLModel(XGT_fit)

print(XG_tuned_model, n = Inf)

#mean(XGT_res$Observed)

XGT_VImp = varimp(XGT_fit)

XGT_calib = calibration(XGT_res)

#XGT_Pdep = dependence(XGT_fit)

plot(XG_tuned_model, type = "line")

plot(XGT_VImp)

#plot(XGT_Pdep)

plot(XGT_calib, breaks = NULL)

```

```{r}

################# Comparing all 4 models ######################

## Compare resampled results

res <- c(GLM = glms_res, GBM = GBM_res, LASSO = lasso_res, XGBT = XGT_res)

summary(res)

plot(res)

```

######Discretization and dichotomization of the predicted and observed values from Lasso regression to generate classification parameters#########

```{r}

## creating discrete variables from the observed and predicted values from Lasso regression (best performing model)

summary(lasso_res$Observed)

lasso_res$Observed

summary(lasso_res$Predicted)

lasso_res$Predicted

lasso_res$Observed.hat<- sapply(lasso_res$Observed, function(l){

(0:100)[which.max(dpois(0:100, lambda = l))]

})

lasso_res$Observed.hat

lasso_res$Predicted.hat<- sapply(lasso_res$Predicted, function(l){

(0:100)[which.max(dpois(0:100, lambda = l))]

})

lasso_res$Predicted.hat

##Dichotomizing the values from the selected model (Lasso regression model).

lasso_res$Observed.hat<- as.factor(ifelse(lasso_res$Observed.hat==0,"NC","D"))

lasso_res$Observed.hat

table(lasso_res$Observed.hat)

lasso_res$Predicted.hat<- as.factor(ifelse(lasso_res$Predicted.hat==0,"NC", "D"))

lasso_res$Predicted.hat

table(lasso_res$Predicted.hat)

## performance assessment

performance(lasso_res$Observed.hat, lasso_res$Predicted.hat)

performance(lasso_res$Observed.hat, lasso_res$Predicted.hat, metrics = c(accuracy, precision, recall))

confusion(lasso_res$Observed.hat, lasso_res$Predicted.hat)

summary(confusion(lasso_res$Observed.hat, lasso_res$Predicted.hat))

## performance assessment

auc(Obs, Pred)

roc <- performance_curve(Obs, Pred)

plot(roc)

plot(roc, type = "cutoffs")

auc(roc)

lasso_fit$beta

```

**Appendix XI: TRIPOD Checklist: Prediction Model Development**

| **Section/Topic** | **Item** | **Checklist Item** | **Page** |
| --- | --- | --- | --- |
| **Title and abstract** | | | |
| Title  **COMPLETED** | 1 | Identify the study as developing and/or validating a multivariable prediction model, the target population, and the outcome to be predicted. |  |
| Abstract  **COMPLETED** | 2 | Provide a summary of objectives, study design, setting, participants, sample size, predictors, outcome, statistical analysis, results, and conclusions. |  |
| **Introduction** | | | |
| Background and objectives  **COMPLETED** | 3a | Explain the medical context (including whether diagnostic or prognostic) and rationale for developing or validating the multivariable prediction model, including references to existing models. | 1 |
|  | 3b | Specify the objectives, including whether the study describes the development or validation of the model or both. | 2 |
| **Methods** | | | |
| Source of data  **COMPLETED** | 4a | Describe the study design or source of data (e.g., randomized trial, cohort, or registry data), separately for the development and validation data sets, if applicable. | 2 |
|  | 4b | Specify the key study dates, including start of accrual; end of accrual; and, if applicable, end of follow-up. | 2 |
| Participants  **COMPLETED** | 5a | Specify key elements of the study setting (e.g., primary care, secondary care, general population) including number and location of centres. | 2 |
|  | 5b | Describe eligibility criteria for participants. | 2 |
|  | 5c | Give details of treatments received, if relevant. | 3 |
| Outcome  **COMPLETED** | 6a | Clearly define the outcome that is predicted by the prediction model, including how and when assessed. | 3 |
|  | 6b | Report any actions to blind assessment of the outcome to be predicted. |  |
| Predictors  **COMPLETED** | 7a | Clearly define all predictors used in developing or validating the multivariable prediction model, including how and when they were measured. | 3 |
|  | 7b | Report any actions to blind assessment of predictors for the outcome and other predictors. | 4 |
| Sample size  **COMPLETED** | 8 | Explain how the study size was arrived at. | 3 |
| Missing data  **COMPLETED** | 9 | Describe how missing data were handled (e.g., complete-case analysis, single imputation, multiple imputation) with details of any imputation method. | 4 |
| Statistical analysis methods  **COMPLETED** | 10a | Describe how predictors were handled in the analyses. | 4 |
|  | 10b | Specify type of model, all model-building procedures (including any predictor selection), and method for internal validation. | 4 |
|  | 10d | Specify all measures used to assess model performance and, if relevant, to compare multiple models. | 4 |
| Risk groups  **COMPLETED** | 11 | Provide details on how risk groups were created, if done. | 4 |
| **Results** | | | |
| Participants  **COMPLETED** | 13a | Describe the flow of participants through the study, including the number of participants with and without the outcome and, if applicable, a summary of the follow-up time. A diagram may be helpful. | 5 |
|  | 13b | Describe the characteristics of the participants (basic demographics, clinical features, available predictors), including the number of participants with missing data for predictors and outcome. | 5 |
| Model development  **COMPLETED** | 14a | Specify the number of participants and outcome events in each analysis. | 5 |
|  | 14b | If done, report the unadjusted association between each candidate predictor and outcome. | 5 |
| Model specification  **COMPLETED** | 15a | Present the full prediction model to allow predictions for individuals (i.e., all regression coefficients, and model intercept or baseline survival at a given time point). | 6 |
|  | 15b | Explain how to the use the prediction model. | 6 |
| Model performance  **COMPLETED** | 16 | Report performance measures (with CIs) for the prediction model. | 6 |
| **Discussion** | | | |
| Limitations  **COMPLETED** | 18 | Discuss any limitations of the study (such as nonrepresentative sample, few events per predictor, missing data). | 8 |
| Interpretation  **COMPLETED** | 19b | Give an overall interpretation of the results, considering objectives, limitations, and results from similar studies, and other relevant evidence. | 7 |
| Implications  **COMPLETED** | 20 | Discuss the potential clinical use of the model and implications for future research. | 8 |
| **Other information** | | | |
| Supplementary information  **COMPLETED** | 21 | Provide information about the availability of supplementary resources, such as study protocol, Web calculator, and data sets. | Sup |
| Funding  **COMPLETED** | 22 | Give the source of funding and the role of the funders for the present study. | Sup |
